# Crystal structure of 2-(3-nitro­phen­yl)-1,3-di­thiane

**DOI:** 10.1107/S2056989015002844

**Published:** 2015-02-13

**Authors:** Ignez Caracelli, Julio Zukerman-Schpector, Hélio A. Stefani, Olga Gozhina, Edward R. T. Tiekink

**Affiliations:** a Departmento de Física, Universidade Federal de São Carlos, 13565-905 São Carlos, SP, Brazil; b Departmento de Química, Universidade Federal de São Carlos, 13565-905 São Carlos, SP, Brazil; c Departamento de Farmácia, Faculdade de Ciências Farmacêuticas, Universidade de São Paulo, 05508-900 São Paulo-SP, Brazil; d Department of Chemistry, University of Malaya, 50603 Kuala Lumpur, Malaysia

**Keywords:** crystal structure, 1,3-di­thiane, conformation, N—O⋯π inter­actions

## Abstract

In the title compound, C_10_H_11_NO_2_S_2_, the 1,3-di­thiane ring has a chair conformation with the 1,4-disposed C atoms being above and below the remaining four atoms. The nitro­benzene substituent occupies an equatorial position and forms a dihedral angle of 88.28 (5)° with the least-squares plane through the 1,3-di­thiane ring. The nitro group is twisted out of the plane of the benzene ring to which it is connected, forming a dihedral angle of 10.12 (3)°. In the crystal, mol­ecules aggregate into supra­molecular zigzag chains (glide symmetry along the *c* axis) *via* nitro–benzene N—O⋯π [N—O⋯*Cg*(benzene) = 3.4279 (18) Å and angle at O = 93.95 (11)°] inter­actions. The chains pack with no specific inter­molecular inter­actions between them.

## Related literature   

For background to substituted 1,3-di­thia­nes, see: Ballesteros *et al.* (2005 ▸). For nitro–aryl N—O⋯π inter­actions, see: Huang *et al.* (2008 ▸). For the structure of the closely related 3-bromo-substituted compound, see: Zukerman-Schpector *et al.* (2015 ▸).
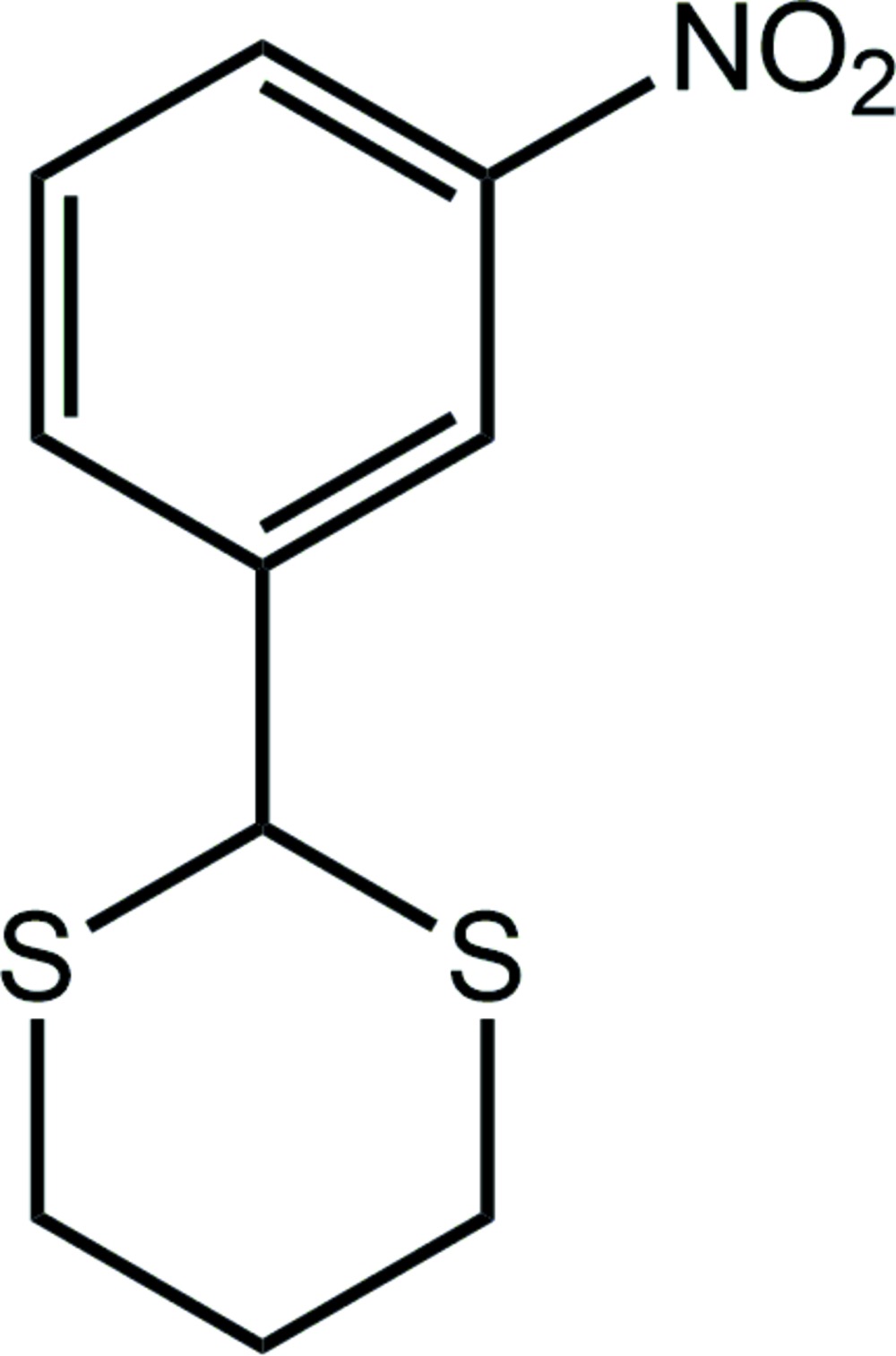


## Experimental   

### Crystal data  

C_10_H_11_NO_2_S_2_
*M* *_r_* = 241.32
Monoclinic, 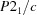

*a* = 10.8547 (2) Å
*b* = 13.2655 (3) Å
*c* = 8.0891 (2) Å
β = 109.087 (1)°
*V* = 1100.74 (4) Å^3^
*Z* = 4
Mo *K*α radiation
μ = 0.46 mm^−1^
*T* = 293 K
0.49 × 0.46 × 0.21 mm

### Data collection  

Bruker APEXII CCD diffractometer
Absorption correction: multi-scan (*SADABS*; Sheldrick, 1996 ▸) *T*_min_ = 0.687, *T*_max_ = 0.745
7241 measured reflections
2035 independent reflections
1799 reflections with *I* > 2σ(*I*)
*R*_int_ = 0.020

### Refinement  

*R*[*F* ^2^ > 2σ(*F* ^2^)] = 0.031
*wR*(*F* ^2^) = 0.089
*S* = 1.06
2035 reflections
137 parameters
H-atom parameters constrained
Δρ_max_ = 0.29 e Å^−3^
Δρ_min_ = −0.24 e Å^−3^

### 

Data collection: *APEX2* (Bruker, 2009 ▸); cell refinement: *SAINT* (Bruker, 2009 ▸); data reduction: *SAINT*; program(s) used to solve structure: *SIR2014* (Burla *et al.*, 2015 ▸); program(s) used to refine structure: *SHELXL2014* (Sheldrick, 2015 ▸); molecular graphics: *ORTEP-3 for Windows* (Farrugia, 2012 ▸) and *DIAMOND* (Brandenburg, 2006 ▸); software used to prepare material for publication: *MarvinSketch* (ChemAxon, 2010 ▸) and *publCIF* (Westrip, 2010 ▸).

## Supplementary Material

Crystal structure: contains datablock(s) I, New_Global_Publ_Block. DOI: 10.1107/S2056989015002844/hg5430sup1.cif

Structure factors: contains datablock(s) I. DOI: 10.1107/S2056989015002844/hg5430Isup2.hkl

Supporting information file. DOI: 10.1107/S2056989015002844/hg5430Isup3.cml

**Fig. 1.**
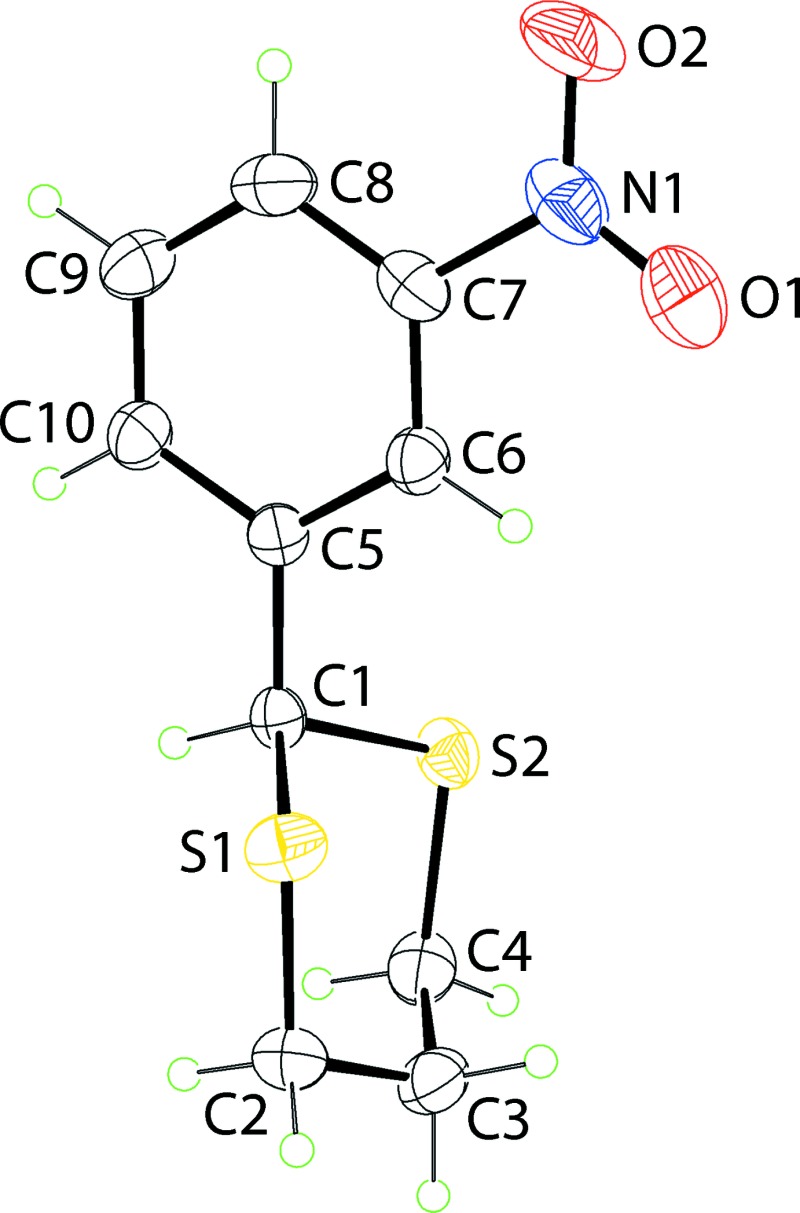
The molecular structure of the title compound showing the atom-labelling scheme and displacement ellipsoids at the 35% probability level.

**Fig. 2.**
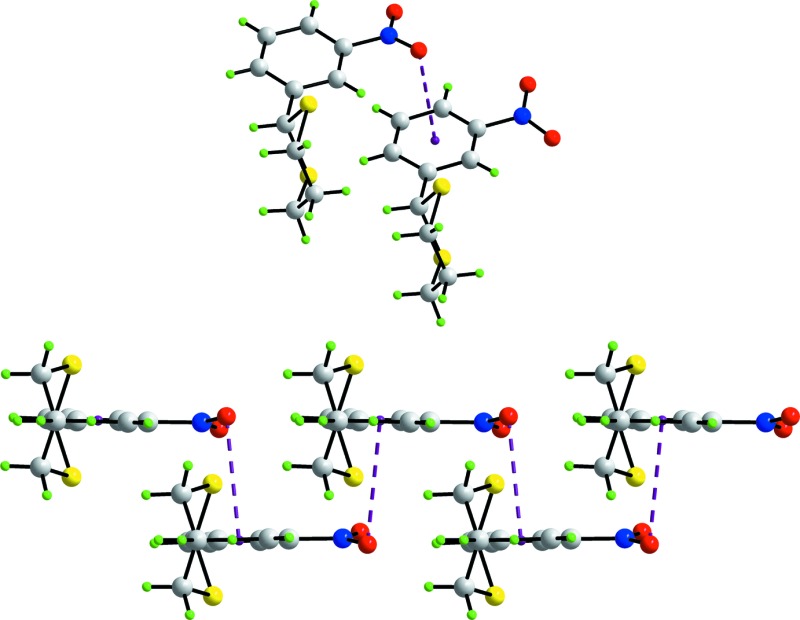
Upper view: detail of the nitro-N—O···π(benzene) interaction. Lower view: the zigzag supramolecular chain along the c axis (glide symmetry) mediated by nitro-N—O···π(benzene) interactions shown as purple dashed lines.

**Fig. 3.**
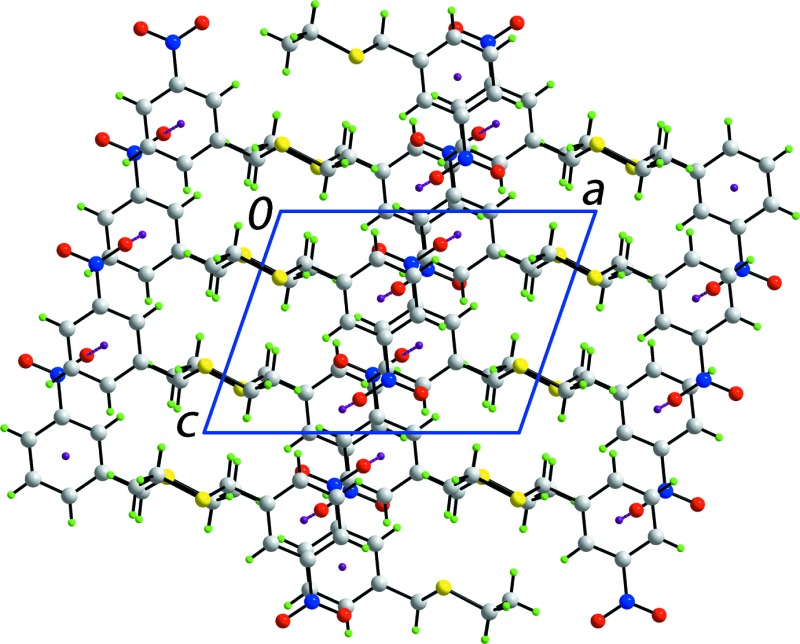
A view in projection down the b axis of the unit-cell contents. The nitro-N—O···π(benzene) interactions are shown as purple dashed lines.

. DOI: 10.1107/S2056989015002844/hg5430fig1.tif
The mol­ecular structure of the title compound showing the atom-labelling scheme and displacement ellipsoids at the 35% probability level.

c . DOI: 10.1107/S2056989015002844/hg5430fig2.tif
Upper view: detail of the nitro-N—O⋯π(benzene) inter­action. Lower view: the zigzag supra­molecular chain along the *c* axis (glide symmetry) mediated by nitro-N—O⋯π(benzene) inter­actions shown as purple dashed lines.

b . DOI: 10.1107/S2056989015002844/hg5430fig3.tif
A view in projection down the *b* axis of the unit-cell contents. The nitro-N—O⋯π(benzene) inter­actions are shown as purple dashed lines.

CCDC reference: 1048518

Additional supporting information:  crystallographic information; 3D view; checkCIF report

## supplementary crystallographic information

### S1. Experimental

A solution of 3-nitrobenzaldehyde (0.037 mol, 1 equiv.) in chloroform (20 ml) was combined with an equimolar amount of propane-1,3-dithiol (3.7 ml, 0.037 mol) at room temperature. The solution was stirred for 1 h at this temperature, then cooled to -20 °C after which BF_3_ etherate (0.46 ml, 0.0037 mol, 0.1 equiv.) was added drop-wise. The reaction solution was allowed to warm to room temperature and stirred overnight. After this time, the solution was washed three times each with water, 10% aqueous KOH, then water followed by drying over MgSO_4_. Evaporation of the solvent furnishes a pure product as colourless crystals in 85% yield. To obtain crystals suitable for X-ray analysis, the product was crystallized from CH_3_OH. ^1^H NMR (300 MHz, CDCl_3_) δ 8.37 (s, 1H), 8.18 (dt, J = 8.3, 2.6 Hz, 1H), 7.83 (ddd, J = 7.2, 3.6, 1.7 Hz, 1H), 7.63–7.48 (m, 1H), 5.26 (s, 1H), 3.10 (ddq, J = 14.4, 12.0, 2.3 Hz, 2H), 2.96 (ddp, J = 13.4, 5.1, 2.7 Hz, 2H), 2.23 (dtq, J = 14.0, 4.6, 2.3 Hz, 1H), 1.98 (dddd, J = 14.3, 12.1, 9.8, 6.0 Hz, 1H). ^13^C NMR (75 MHz, CDCl_3_) δ 148.39, 141.18, 133.99, 129.70, 123.36, 123.12, 50.19 (2 *x* C), 31.75, 24.78. *M*.pt: 368 K. IR (cm^-1^): ν 1525 (N—O); 1348 (N—O); 724 and 687 (C—S),

### S2. Refinement

Carbon-bound H-atoms were placed in calculated positions (C—H = 0.93–0.98 Å) and were included in the refinement in the riding model approximation, with *U*_iso_(H) = 1.2 *U*_eq_(C).

### Crystal data

**Table:**

C_10_H_11_NO_2_S_2_	*F*(000) = 504
*M**_r_* = 241.32	*D*_x_ = 1.456 Mg m^−^^3^
Monoclinic, *P*2_1_/*c*	Mo *K*α radiation, λ = 0.71073 Å
*a* = 10.8547 (2) Å	Cell parameters from 4425 reflections
*b* = 13.2655 (3) Å	θ = 2.5–25.4°
*c* = 8.0891 (2) Å	µ = 0.46 mm^−^^1^
β = 109.087 (1)°	*T* = 293 K
*V* = 1100.74 (4) Å^3^	Slab, colourless
*Z* = 4	0.49 × 0.46 × 0.21 mm

### Data collection

**Table:**

Bruker APEXII CCD diffractometer	1799 reflections with *I* > 2σ(*I*)
φ and ω scans	*R*_int_ = 0.020
Absorption correction: multi-scan (*SADABS*; Sheldrick, 1996)	θ_max_ = 25.4°, θ_min_ = 2.0°
*T*_min_ = 0.687, *T*_max_ = 0.745	*h* = −13→12
7241 measured reflections	*k* = −16→16
2035 independent reflections	*l* = −8→9

### Refinement

**Table:**

Refinement on *F*^2^	Hydrogen site location: inferred from neighbouring sites
Least-squares matrix: full	H-atom parameters constrained
*R*[*F*^2^ > 2σ(*F*^2^)] = 0.031	*w* = 1/[σ^2^(*F*_o_^2^) + (0.0444*P*)^2^ + 0.3472*P*] where *P* = (*F*_o_^2^ + 2*F*_c_^2^)/3
*wR*(*F*^2^) = 0.089	(Δ/σ)_max_ < 0.001
*S* = 1.06	Δρ_max_ = 0.29 e Å^−^^3^
2035 reflections	Δρ_min_ = −0.24 e Å^−^^3^
137 parameters	Extinction correction: *SHELXL2014* (Sheldrick 2014, Fc^*^=kFc[1+0.001xFc^2^λ^3^/sin(2θ)]^-1/4^
0 restraints	Extinction coefficient: 0.0192 (17)

### Special details

**Table:**

Geometry. All e.s.d.'s (except the e.s.d. in the dihedral angle between two l.s. planes) are estimated using the full covariance matrix. The cell e.s.d.'s are taken into account individually in the estimation of e.s.d.'s in distances, angles and torsion angles; correlations between e.s.d.'s in cell parameters are only used when they are defined by crystal symmetry. An approximate (isotropic) treatment of cell e.s.d.'s is used for estimating e.s.d.'s involving l.s. planes.

### Fractional atomic coordinates and isotropic or equivalent isotropic displacement parameters (Å^2^)

**Table:**

	*x*	*y*	*z*	*U*_iso_*/*U*_eq_	
S1	0.93087 (4)	0.75719 (3)	0.69969 (5)	0.04460 (16)	
S2	0.93019 (4)	0.98623 (3)	0.69835 (6)	0.04646 (16)	
O1	0.54267 (18)	0.88063 (13)	0.15045 (19)	0.0830 (5)	
O2	0.35484 (16)	0.85594 (14)	0.1765 (2)	0.0917 (6)	
N1	0.47188 (18)	0.86855 (11)	0.2380 (2)	0.0608 (5)	
C1	0.86732 (14)	0.87164 (10)	0.76325 (19)	0.0340 (3)	
H1	0.8887	0.8719	0.8907	0.041*	
C2	1.10282 (17)	0.96790 (14)	0.8068 (2)	0.0511 (4)	
H2A	1.1494	1.0248	0.7804	0.061*	
H2B	1.1198	0.9673	0.9322	0.061*	
C3	1.15622 (17)	0.87200 (13)	0.7556 (2)	0.0519 (5)	
H3A	1.1364	0.8713	0.6297	0.062*	
H3B	1.2503	0.8720	0.8085	0.062*	
C4	1.10278 (17)	0.77709 (14)	0.8096 (2)	0.0503 (4)	
H4A	1.1185	0.7796	0.9347	0.060*	
H4B	1.1503	0.7198	0.7868	0.060*	
C5	0.72187 (15)	0.87186 (10)	0.6807 (2)	0.0353 (3)	
C6	0.66533 (16)	0.86907 (11)	0.4999 (2)	0.0403 (4)	
H6	0.7169	0.8669	0.4280	0.048*	
C7	0.53161 (17)	0.86960 (11)	0.4295 (2)	0.0454 (4)	
C8	0.45093 (17)	0.87258 (13)	0.5303 (3)	0.0538 (5)	
H8	0.3607	0.8722	0.4790	0.065*	
C9	0.50821 (18)	0.87617 (13)	0.7092 (3)	0.0539 (5)	
H9	0.4561	0.8788	0.7803	0.065*	
C10	0.64180 (17)	0.87591 (11)	0.7842 (2)	0.0441 (4)	
H10	0.6789	0.8785	0.9053	0.053*	

### Atomic displacement parameters (Å^2^)

**Table:**

	*U*^11^	*U*^22^	*U*^33^	*U*^12^	*U*^13^	*U*^23^
S1	0.0499 (3)	0.0379 (2)	0.0436 (3)	0.00382 (16)	0.01201 (19)	−0.00589 (15)
S2	0.0441 (3)	0.0374 (3)	0.0589 (3)	−0.00061 (16)	0.0181 (2)	0.00780 (17)
O1	0.0889 (12)	0.1119 (14)	0.0382 (8)	0.0027 (9)	0.0071 (8)	0.0037 (7)
O2	0.0563 (9)	0.1212 (14)	0.0696 (10)	0.0056 (9)	−0.0178 (8)	−0.0135 (9)
N1	0.0629 (11)	0.0593 (10)	0.0454 (9)	0.0066 (7)	−0.0023 (8)	−0.0033 (7)
C1	0.0388 (8)	0.0344 (8)	0.0294 (7)	−0.0002 (6)	0.0119 (6)	−0.0002 (5)
C2	0.0416 (9)	0.0581 (11)	0.0532 (10)	−0.0114 (8)	0.0152 (8)	−0.0059 (8)
C3	0.0368 (8)	0.0729 (13)	0.0477 (10)	0.0044 (8)	0.0161 (7)	0.0004 (8)
C4	0.0467 (9)	0.0580 (11)	0.0433 (9)	0.0163 (8)	0.0107 (7)	0.0050 (7)
C5	0.0378 (8)	0.0338 (8)	0.0351 (8)	−0.0015 (6)	0.0128 (6)	0.0003 (5)
C6	0.0426 (9)	0.0438 (9)	0.0355 (8)	0.0009 (6)	0.0140 (7)	0.0009 (6)
C7	0.0456 (9)	0.0417 (9)	0.0408 (9)	−0.0002 (7)	0.0032 (7)	−0.0022 (6)
C8	0.0353 (9)	0.0542 (11)	0.0689 (12)	−0.0036 (7)	0.0131 (8)	−0.0020 (8)
C9	0.0462 (10)	0.0625 (12)	0.0608 (11)	−0.0065 (8)	0.0282 (9)	−0.0023 (8)
C10	0.0472 (9)	0.0490 (9)	0.0403 (9)	−0.0052 (7)	0.0201 (7)	−0.0006 (7)

### Geometric parameters (Å, º)

**Table:**

S1—C4	1.8048 (18)	C3—H3B	0.9700
S1—C1	1.8102 (14)	C4—H4A	0.9700
S2—C2	1.8069 (18)	C4—H4B	0.9700
S2—C1	1.8128 (14)	C5—C6	1.389 (2)
O1—N1	1.214 (2)	C5—C10	1.391 (2)
O2—N1	1.215 (2)	C6—C7	1.375 (2)
N1—C7	1.471 (2)	C6—H6	0.9300
C1—C5	1.500 (2)	C7—C8	1.379 (3)
C1—H1	0.9800	C8—C9	1.377 (3)
C2—C3	1.511 (2)	C8—H8	0.9300
C2—H2A	0.9700	C9—C10	1.377 (3)
C2—H2B	0.9700	C9—H9	0.9300
C3—C4	1.509 (3)	C10—H10	0.9300
C3—H3A	0.9700		
			
C4—S1—C1	99.55 (8)	C3—C4—H4A	108.7
C2—S2—C1	100.11 (8)	S1—C4—H4A	108.7
O1—N1—O2	123.77 (18)	C3—C4—H4B	108.7
O1—N1—C7	117.90 (17)	S1—C4—H4B	108.7
O2—N1—C7	118.32 (19)	H4A—C4—H4B	107.6
C5—C1—S1	108.59 (10)	C6—C5—C10	119.13 (15)
C5—C1—S2	108.10 (10)	C6—C5—C1	120.46 (13)
S1—C1—S2	113.99 (8)	C10—C5—C1	120.41 (14)
C5—C1—H1	108.7	C7—C6—C5	118.65 (15)
S1—C1—H1	108.7	C7—C6—H6	120.7
S2—C1—H1	108.7	C5—C6—H6	120.7
C3—C2—S2	114.22 (12)	C6—C7—C8	122.93 (16)
C3—C2—H2A	108.7	C6—C7—N1	118.60 (16)
S2—C2—H2A	108.7	C8—C7—N1	118.46 (16)
C3—C2—H2B	108.7	C9—C8—C7	117.84 (16)
S2—C2—H2B	108.7	C9—C8—H8	121.1
H2A—C2—H2B	107.6	C7—C8—H8	121.1
C4—C3—C2	113.89 (15)	C10—C9—C8	120.77 (16)
C4—C3—H3A	108.8	C10—C9—H9	119.6
C2—C3—H3A	108.8	C8—C9—H9	119.6
C4—C3—H3B	108.8	C9—C10—C5	120.67 (16)
C2—C3—H3B	108.8	C9—C10—H10	119.7
H3A—C3—H3B	107.7	C5—C10—H10	119.7
C3—C4—S1	114.37 (12)		
			
C4—S1—C1—C5	178.82 (10)	C1—C5—C6—C7	−179.86 (13)
C4—S1—C1—S2	58.27 (10)	C5—C6—C7—C8	−0.1 (2)
C2—S2—C1—C5	−178.78 (10)	C5—C6—C7—N1	178.88 (13)
C2—S2—C1—S1	−57.95 (10)	O1—N1—C7—C6	−9.5 (2)
C1—S2—C2—C3	57.35 (14)	O2—N1—C7—C6	170.67 (16)
S2—C2—C3—C4	−65.25 (19)	O1—N1—C7—C8	169.54 (17)
C2—C3—C4—S1	66.18 (19)	O2—N1—C7—C8	−10.3 (2)
C1—S1—C4—C3	−58.63 (14)	C6—C7—C8—C9	0.7 (2)
S1—C1—C5—C6	−60.31 (15)	N1—C7—C8—C9	−178.35 (15)
S2—C1—C5—C6	63.82 (15)	C7—C8—C9—C10	−0.5 (3)
S1—C1—C5—C10	120.35 (13)	C8—C9—C10—C5	−0.1 (2)
S2—C1—C5—C10	−115.51 (13)	C6—C5—C10—C9	0.6 (2)
C10—C5—C6—C7	−0.5 (2)	C1—C5—C10—C9	179.98 (14)

## References

[bb1] Ballesteros, L., Noguez, O., Arroyo, G., Velasco, B., Delgado, F. & Miranda, R. (2005). *J. Mex. Chem. Soc* **49**, 302–306.

[bb2] Brandenburg, K. (2006). *DIAMOND*. Crystal Impact GbR, Bonn, Germany.

[bb3] Bruker (2009). *APEX2* and *SAINT*. Bruker AXS Inc., Madison, Wisconsin, USA.

[bb4] Burla, M. C., Caliandro, R., Carrozzini, B., Cascarano, G. L., Cuocci, C., Giacovazzo, C., Mallamo, M., Mazzone, A. & Polidori, G. (2015). *J. Appl. Cryst.* **48**, 306–309.

[bb5] ChemAxon (2010). *Marvinsketch*. http://www.chemaxon.com.

[bb6] Farrugia, L. J. (2012). *J. Appl. Cryst.* **45**, 849–854.

[bb7] Huang, L., Massa, L. & Karle, J. (2008). *Proc. Natl Acad. Sci.* **105**, 13720–13723. 10.1073/pnas.0807218105 PMC2544520 18780785

[bb8] Sheldrick, G. M. (1996). *SADABS*. University of Göttingen, Germany.

[bb9] Sheldrick, G. M. (2015). *Acta Cryst.* C**71**, 3–8.

[bb10] Westrip, S. P. (2010). *J. Appl. Cryst.* **43**, 920–925.

[bb11] Zukerman-Schpector, J., Caracelli, I., Stefani, H. A., Gozhina, O. & Tiekink, E. R. T. (2015). *Acta Cryst.* E**71**, o179–o180. 10.1107/S2056989015002832 PMC4350742 25844236

